# Efficacy of acupuncture in subpopulations with functional constipation: A protocol for a systematic review and individual patient data meta-analysis

**DOI:** 10.1371/journal.pone.0266075

**Published:** 2022-04-12

**Authors:** Chao Chen, Jia Liu, Baoyan Liu, Xue Cao, Zhishun Liu, Tianyi Zhao, Xiaoying Lv, Shengnan Guo, Yang Li, Liyun He, Yanke Ai

**Affiliations:** 1 Institute of Basic Research for Clinical Medicine, China Academy of Chinese Medical Sciences, Beijing, China; 2 China Astronaut Research and Training Center, Beijing, China; 3 China Academy of Chinese Medical Sciences, Beijing, China; 4 Guang’an Men Hospital, China Academy of Chinese Medical Sciences, Beijing, China; 5 Institute of Acupuncture and Moxibustion, China Academy of Chinese Medical Sciences, Beijing, China; 6 Beijing Fengtai Youanmen Hospital, Beijing, China; International Hellenic University: Diethnes Panepistemio tes Ellados, GREECE

## Abstract

**Background:**

Several systematic reviews have suggested that acupuncture is effective against functional constipation, but it is unknown whether variations in treatment effect across subgroups remain consistent. Our purpose of this study is to explore the heterogeneity of treatment effect of acupuncture on functional constipation across subgroups.

**Methods:**

We will search eleven English and Chinese electronic databases and three clinical trial registries from inception to December 2021. Randomized controlled trials that evaluate acupuncture compared with sham acupuncture or no treatment for functional constipation will be eligible if they report at least one primary outcome. The primary outcomes will include the change in weekly complete spontaneous bowel movements or spontaneous bowel movements from baseline. Two authors will independently identify the relevant studies, assess the risk of bias using the Cochrane RoB 2 tool and contact the primary researchers of the eligible trials for individual patient data. Individual patient data obtained from the original trial author will be standardized and all trial data will be combined into a single database. Generalized linear mixed effects model will be used to determine possible subgroup effects by adding an interaction term for predefined subgroup and treatment.

**Systematic review registration:**

International Prospective Register of Systematic Reviews (Number: CRD42020188366).

## Introduction

Functional constipation is a group of functional bowel disorder, in which patients have symptoms of persistent, difficult, seemingly incomplete defecation or infrequent bowel movements [1]. It is reported that about 10–15% of the global population is affected by functional constipation [2] and certain demographic groups, such as women [3], the elderly [4], high body mass index [5], low socioeconomic status or educational level [6, 7], family history of constipation [8] are more prone to constipation. Although the symptoms associated with constipation are often intermittent and mild, they may be chronic, difficult to treat, and even debilitating [9], which affects quality of life and results in a major social and economic burden [10].

Due to a lack of efficacy, approximately half of the patients were not satisfied with the current treatments, including laxatives and dietary fiber [11]. The efficacy of acupuncture for functional constipation has been confirmed by one multicenter, randomized, sham-controlled trial [12] and several systematic reviews and meta-analyses [13–15] based on aggregate patient data (APD). However, these studies mainly focused on the effectiveness in the general patient population, and few studies have assessed whether the treatment effect of acupuncture remain consistent across patients with different characteristics. Significant treatment effects in relevant characteristic populations may be masked in the analysis of the total patient population. Determining whether the observed overall treatment effect is different across certain subgroups may provide greater assurance for patients, benefiting them while protecting them from its harm [16].

Individual patient data (IPD) meta-analysis is the gold standard of systematic review. Compared with APD meta-analysis, which is reliant on extracting summary results for each subgroup and often not be available, IPD meta-analysis can be conducted more powerfully and flexibly for subgroup patients with a common characteristic [17, 18]. Therefore, the purpose of this study was to use a one-step IPD meta-analysis to explore heterogeneity of treatment effect of acupuncture on functional constipation across subgroups, and found evidence for the existence of subgroups of patients with functional constipation would be more likely to benefit from acupuncture.

## Methods and analysis

This protocol is reported in accordance with the Preferred Reporting Items for Systematic Reviews and Meta-Analyses of individual participant data (PRISMA-IPD) 2015 Statement [19].

The Protocol is developed under the guidance of China Academy of Chinese Medical Sciences (CACMS) ClinResearch Trialists’ collaboration. This collaboration is a working group of researchers including the clinical acupuncturists, statisticians, and methodologists at the Clinical Evaluation Center of CACMS. Each member of collaboration was fully involved in the design of the protocol. The collaboration will also include the primary investigators that share the IPD.

The CACMS CliniRsearch Data Management System (available from http://118.144.35.11/wcr/) was established at CACMS to serve as the platform for clinical research data capturing and now contains the raw data of over 260 clinical trials, most of which are supported by the Chinese government. We have already obtained the researchers’ permission to obtain access to the IPD of four related trials for a total of 1782 IPD, one of which has been published [12], and three earlier exploratory studies have not been published.

### Patient and public involvement

No patient involved in the development of the protocol.

### Inclusion and exclusion criteria for considering studies

Studies will be selected according to the following criteria.

#### Study type

We will include only randomized controlled trials (RCTs). To ensure the quality of the data, we will include only the RCTs that have a low risk of bias (RoB 2) in the randomization process, which means they are free of bias in random sequence generation and allocation concealment. Language will be restricted to English and Chinese.

#### Patients

We will include the patients diagnosed of functional constipation. The diagnosis of functional constipation should be based on Rome II [20]/III [21]/IV [22] diagnostic criteria and other approved guidelines, such as European Society of Neurogastroenterology and Motility (ESNM) 2019 [23] and American Gastroenterological Association [24]. Constipation secondary to another underlying disorder, such as medication, anatomical alterations, neurologic diseases, or metabolic disturbances, will be excluded.

#### Interventions

We will include relevant RCTs using acupuncture for at least 2 weeks. As acupuncture refers to a family of procedures that stimulate anatomical locations on the body by a variety of techniques [25]. We will not limit the stimulation techniques, which could include manual needles, electroacupuncture, lasers needle, acupoint injection, or other sources of stimulation.

The control group received sham acupuncture or no acupuncture will be eligible. Sham acupuncture is defined as any intervention designed to prevent the patients from knowing whether he or she has received real acupuncture, which includes needling at non-acupoints, non-penetrating needling or the use of placebo needles such as Streitberger and Kleinhenz needles [26].

In addition, combined treatments should be the same between the groups. The combined treatments may include lifestyle interventions, dietary suggestions, health education and necessary laxative agents.

#### Outcomes

We will include the RCTs which at least assessed the outcome of weekly complete spontaneous bowel movements (CSBM) or spontaneous bowel movement (SBM). Primary outcomes are the change in CSBM/SBM from baseline. SBM is defined as a bowel movement without the use of any medication or other methods to assist defecation in the previous 24 hours, and a CSBM is defined as an SBM associated with a sensation of complete evacuation [27].

Secondary outcomes include the Patient Assessment of Constipation Quality of Life questionnaire (PAC-QOL) [28], the Bristol Stool Form Scale, and general quality of life as measured by various instruments (such as the SF-36 and EQ-5D).

### Search strategies

The following 11 electronic databases will be searched from inception to December 2021 for potential studies: the Cochrane Central Register of Controlled Trials (CENTRAL), MEDLINE, Scopus, Embase, AMED (Allied and Alternative Medicine), Science Citation Index Expanded (Web of Science), Conference Proceedings Citation Index–Science, the China National Knowledge Infrastructure (CNKI), SinoMed, WanFang Data and VIP database. Ongoing trials will be searched in the WHO International Clinical Trials Registry Platform (ICTRP) and ClinicalTrials.gov and Chinese Clinical Trial Registry (ChiCTR). Free text and subject terms related to functional constipation and acupuncture will be used to develop systematic search strategies (see ‘Search strategies’ in S1 File). The references of any identified meta-analyses will be screened as supplementary sources. We will also consult the key authors for additional literature and unpublished trials.

### Data collection and data management

#### Selection of studies

All retrieved references will be imported to Endnote X9 for reference management. After the removal of duplicate references by the software, two independent investigators will select eligible studies among the remaining references by scanning the titles and abstracts according to the inclusion criteria. For each excluded reference, the reasons for exclusion will be given. Disagreements will be resolved by the third reviewer.

#### Risk-of-bias assessment

All the included trials will be assessed by two independent investigators using the revised Cochrane tool for assessing the risk of bias in randomized trials (RoB 2) [29]. The revised RoB tool assesses five domains of bias, comprising the randomization process, deviations from intended interventions, missing outcome data, measurement of the outcome, selection of the reported results. All the domains will be rated with either ‘low risk of bias’, ‘some concerns’, or ‘high risk of bias’ based on the signalling questions. Disagreements will be resolved by consensus.

#### Acquisition and transfer of the IPD

IPD from four trials are available from the CACMS CliniRsearch Data Management System. For the other trials, we will contact the corresponding authors of the included studies by E-mail to invite them to participate in our collaboration and provide the IPD. If there is no response from the authors after 2 weeks, we will send a reminder. We will send a maximum of three reminders to each author before marking the author as not responding. If there is no response, we will try to contact other authors of the trial. If none of the authors respond or one of the authors claim that the group is unwilling to share the data, the IPD of the study will marked unavailable. If the authors are willing to participate in our collaboration and agree to share data, we will sign a data share agreement. The authors will also be invited to be co-authors of the systematic review and be asked to share the relevant suggestion for the analysis. Any change from the protocol will be documented.

The key variables of patient-level data we plan to collect are as follows: 1) general information of trial: the number of participants, the number and name of sites, method and schemes of randomization, detailed description of intervention/control, the number or duration of treatment the trial planed, the visit time frame of the trial, etc. 2) demographic characteristics: age-related information, diagnosis information related to functional constipation, course of disease, subtypes of functional constipation, sex, nationality, education background, occupation, body mass index (BMI), and family history of constipation etc. 3) compliance of treatment: the number of actual treatments the patient received. 4) outcomes variables: CSBM, SBM, Bristol score, PAC-QOL, etc.

Data set will be accepted in any format. In order to protect the subjects’ privacy, we will request the IPD provider that the identifiable patient and sensitive information (such as name, telephone number, address, driver license number and ID number) must be sufficiently anonymized and removed. All de-identified data will be transferred by creating an encrypted zip file.

#### Data management

The raw data will be saved in their original format and then imported to R 4.0.3 and be saved in the RData format. R and the package ‘dplyr’ will be used for data manipulation. Each dataset will be reviewed for the completeness and accuracy, including checking missing and duplicate data, searching for unreasonable values, and identifying the outlier data. In addition, data for each trial will be checked for consistency with the published reports and any discrepancies will be documented. If individual patient-level data are missing or questionable, we will contact the original investigators for help.

#### Data standardization and aggregation

After data management, all data will be standardized and merged into a single database for the final analysis. The following tasks will be performed: renaming and labelling the variables, converting each variable to the appropriate data type (numeric, character or factor) and standardizing different units to the same scale. After coding and standardization for each trial’s raw data have been completed, relevant data of each trial will be copied into a large IPD database, structured by trial and individual patient ID. Two programmers will independently double program to aggregate the IPD database and check the consistency.

### Statistical analysis plan

#### Study populations

For overall treatment effect estimates of the primary outcomes, all the analyses will be based on the intention-to-treat (ITT) principle, which means that the analysis will include all randomized patients, regardless of the treatment they actually received, whether they subsequently withdrew from treatment or whether there were any deviations from the protocol [30, 31].

#### Comparisons of baseline

Demographic and baseline data will be performed to identify the differences between two groups of trials using all combined data. In general, continuous variables will be compared using a two-sample t-test or Wilcoxon rank-sum test if data don’t conform a normal distribution. Categorical data will be compared using chi-square test or Fisher’s exact test.

#### Analyses of primary outcomes

In order to minimize the missing data of primary outcomes, only trials with CSBM or SBM outcome assessment will be included in this IPD meta-analysis (see ‘Inclusion and exclusion Criteria for considering studies’ above). Missing data for CSBM/SBM will be reported (%) and imputed by multiple imputation under the missing-at-random assumption [32].

The analysis will be conducted by a one-stage approach, which means that all IPDs are modelled simultaneously while accounting for the clustering of participants within studies [33]. Generalized linear mixed effects model (GLMM) will be used to analyze the effectiveness of acupuncture on CSBM/SBM, reported as differences in means and 95% confidence interval [34].We will first assess the homogeneity of the treatment effects between trials by including an interaction term between the treatment and trial with trial, treatment and treatment*trial as a fixed effect and reporting the *P* value. As we anticipated that the treatment effect might be affected by predefined subgroup factors. Therefore, we will run the second GLMM to test whether an interaction of the treatment and the predefined subgroup exist with trail as a random effect and predefined subgroup, treatment, and predefined subgroup * treatment as a fixed effect. Predefined subpopulations will be taken into account as follows: (1) sex (female vs male); (2) age (toddlers (1–3 years); children (4–18 years), adults (19–59 years) and elders (≥ 60 years)) [35–37]; (3) baseline constipation severity (serious (CSBM≤2); not serious (CSBM>2)); (4) job category (mental work, physical work); (5) education background (primary education or less, secondary education, tertiary education); (6) BMI (BMI≥30 kg/m^2^, BMI<30 kg/m^2^); (7) Acupuncture characteristics, needling depth at ST25(depth≥20mm, depth<20 mm); (8) Functional constipation subtypes (normal-transit constipation, slow-transit constipation, pelvic floor dysfunction, combination of slow-transit constipation and defecatory disorder) [24, 38]. Because some our pre-determined subgroup factors may not have been measured in each study and are systematically missing in some individual studies, complete-case analysis will be used for subgroup analysis.

Finally, the primary researchers of the included original trials will be invited to provide useful insights into the analysis and the final interpretation of the results.

The quality of evidence for the main outcomes will be evaluated by the Grading of Recommendations Assessment, Development and Evaluation (GRADE) guidelines [39], and a summary of findings (SoF) table will be generated by the GRADEpro Guideline Development Tool (GRADEpro GDT, available from gradepro.org).

#### Analyses of secondary outcomes

The analyses of the secondary endpoints will be the same as the analysis of the primary outcomes. We will find out which functional constipation subpopulation benefit most in acupuncture treatments and find any differences from the primary outcomes.

#### Other prespecified analyses

Some acupuncturists claim that a subset of patients is good ‘responders’ to acupuncture therapy [40]; accordingly, logistic regression of stratified by trial will attempt to identify the specific factors that might influence treatment. The dependent variable will be defined as ‘good responders’ (50% or greater increase in CSBM from baseline) or ‘non-responders’ (less than 50% increase in CSBM from baseline). The independent variables will include intervention (acupuncture vs sham acupuncture), age, baseline CSBM/SBM, sex and other baseline covariates.

#### Sensitivity analysis

Since compliance with acupuncture has an impact on treatment outcomes, we plan to perform sensitivity analysis by excluding patients with poor adherence to acupuncture (<80%), and any missing CSBM/SBM data will be imputed using the last observation carried forward (LOCF) method. In addition, because the different cutoff points of subgroups may lead to differences in results, the sensitivity analysis will be carried out by changing the subgroup cutoff points, such as changing the age classification to 10-year bins.

#### Publication bias

If the primary outcomes are available for sufficient numbers of included IPD and APD studies (n > 10), publication bias will be assessed by funnel plots and Egger’s test [41]. Significant publication bias is defined as a p-value <0.1 on Egger’s test. If publication bias is detected, the trim-and-fill method will be used to correct for the asymmetry of the funnel plot and estimate the effect of publication bias [42].

*Statistical software*. All analyses will be conducted with the use of SAS software 9.4 (SAS Institute), R 4.0.3 and Stata 15.0 (StataCorp). The ‘lme4’ and ‘nlme’ packages will be used for the one-stage IPD meta-analysis modelling. In Stata, ‘metafor’ and ‘ipdmeta’ packages will be used for the analysis.

## Discussion

In meta-analysis, different baseline characteristics of patients were important factors of explaining the heterogeneity between studies. However, due to the limited information of published literature, APD meta-analysis cannot properly take patient-level characteristics into account for the analysis and is difficult to identify the variation of the treatment effect across clinical subgroups [43]. IPD meta-analysis is the most reliable and often the only way to investigate whether intervention effects vary by participant characteristics and may find which subpopulations benefit most in acupuncture treatment [44]. Additionally, acupuncture is a highly heterogeneous treatment procedure that varies in stimulation type, acupoint selection, needling depth, manipulation technique, etc. Manipulation techniques are quite different across various regions of the world, and IPD meta-analysis may help to explore the predictors of the outcome.

To date, most IPD meta-analyses have used a two-stage approach [45]. Although the one-stage and two-stage approaches usually yield similar estimates of treatment effects [46], one-stage models are preferable in some situations, and their use has increased dramatically in recent years [47]. A one-stage approach can allow for differences between studies by including study-specific effects in the statistical model [48]. In our research, we are interested in studying the interaction between patient characteristics and treatment effects, and a one-stage model is preferred in this situation [49].

Patient-level data are often difficult to obtain from researchers. We are taking two measures to obtain IPD. One of our IPD sources is the CACMS CliniRsearch Data Management System. In addition, we plan to contact authors through two acupuncture associations, namely, the China Association of Acupuncture-Moxibustion and the World Federation of Acupuncture-Moxibustion Societies, as we are in close contact with the secretariats of these two associations. Through these two acupuncture associations, of which one is the largest such group based in China and the other is the largest in the world, we can gain access to acupuncture researchers and gain trust from them.

Finally, this review will provide meta-analysis evidence based upon IPD regarding the difference of treatment effects of acupuncture on functional constipation among different subgroups, and it will explore the factors that might influence treatment effect, which may help to improve the management of functional constipation.

## Supporting information

S1 Checklist PRISMA IPD. (DOCX)

S2 Checklist PRISMA-P 2015 checklist. (DOCX)

S1 File Search strategies. (DOCX)

S1 Appendix Search strategies. (DOCX)
