# Supplementary material for: Efficacy of acupuncture in subpopulations with functional constipation: A protocol for a systematic review and individual patient data meta-analysis
Source: PLoS One. 2022 Apr 12;17(4):e0266075. doi: 10.1371/journal.pone.0266075 (PMC9004736; doi:10.1371/journal.pone.0266075)
Supplement: S1 Checklist — (DOCX) [file pone.0266075.s001.docx]

| **PRISMA-IPD Section/Topic** | **Item No.b** | **Checklist Item** | **Reported on page #** |
| --- | --- | --- | --- |
| **Title** |  |  |  |
| Title |  | Identify the report as a systematic review and meta-analysis of individual participant data. | 1 |
| **Abstract** |  |  |  |
| Structured summary | 2 | Provide a structured summary including as applicable: | 2-3 |
|  |  | Background: state research question and main objectives, with information on participants,interventions, comparators, and outcomes. | 2 |
|  |  | Methods: report eligibility criteria; data sources including dates of last bibliographic search or elicitation, noting that IPD were sought; methods of assessing risk of bias. | 2 |
|  |  | Results: provide number and type of studies and participants identified and number (%) obtained;  summary effect estimates for main outcomes (benefits and harms) with confidence intervals and  measures of statistical heterogeneity. Describe the direction and size of summary effects in terms  meaningful to those who would put findings into practice. | Not applicable |
|  |  | Discussion: state main strengths and limitations of the evidence, general interpretation of the results,  and any important implications. | Not applicable |
|  |  | Other: report primary funding source, registration number, and registry name for the systematic  review and IPD meta-analysis. | Not applicable |
| **Introduction** |  |  |  |
| Rationale | 3 | Describe the rationale for the review in the context of what is already known. | 3-4 |
| Objectives | 4 | Provide an explicit statement of the questions being addressed with reference, as applicable, to participants, interventions, comparisons, outcomes, and study design (PICOS). Include any hypotheses that relate to particular types of participant-level subgroups. | 4 |
| **Methods** |  |  |  |
| Protocol and  registration | 5 | Indicate if a protocol exists and where it can be accessed. If available, provide registration information including registration number and registry name. Provide publication details, if applicable. | 4 |
| Eligibility criteria | 6 | Specify inclusion and exclusion criteria including those relating to participants, interventions,comparisons, outcomes, study design, and characteristics (eg, years when conducted, required minimum follow-up). Note whether these were applied at the study or individual level, ie, whether eligible participants were included (and ineligible participants excluded) from a study that included a wider population than specified by the review inclusion criteria. The rationale for criteria should be stated. | 5-7 |
| Identifying  studies—information  sources | 7 | Describe all methods of identifying published and unpublished studies including, as applicable: which bibliographic databases were searched with dates of coverage; details of any hand searching including of conference proceedings; use of study registers and agency or company databases; contact with the original research team and experts in the field; open advertisements; and surveys. Give the date of last search or elicitation. | 7-8 |
| Identifying  studies—search | 8 | Present the full electronic search strategy for at least 1 database, including any limits used, such that it could be repeated. | 8 |
| Study selection  processes | 9 | State the process for determining which studies were eligible for inclusion. | 8 |
| Data collection  processes | 10 | Describe how IPD were requested, collected, and managed, including any processes for querying and confirming data with investigators. If IPD were not sought from any eligible study, the reason for this  should be stated (for each such study). | 9 |
|  |  | If applicable, describe how any studies for which IPD were not available were dealt with. This should include whether, how, and what aggregate data were sought or extracted from study reports and publications (such as extracting data independently in duplicate) and any processes for obtaining and confirming these data with investigators. | Not applicable |
| Data items | 11 | Describe how the information and variables to be collected were chosen. List and define all study-level and participant-level data that were sought, including baseline and follow-up information. If applicable, describe methods of standardizing or translating variables within the IPD data sets to ensure common scales or measurements across studies. | 10 |
| IPD integrity | A1 | Describe what aspects of IPD were subject to data checking (such as sequence generation, data consistency and completeness, baseline imbalance) and how this was done. | 10-11 |
| Risk of bias assessment  in individual studies | 12 | Describe methods used to assess risk of bias in the individual studies and whether this was applied separately for each outcome. If applicable, describe how findings of IPD checking were used to inform the assessment. Report if and how risk of bias assessment was used in any data synthesis. | 9 |
| Specification of  outcomes and effect  measures | 13 | State all treatment comparisons of interest. State all outcomes addressed and define them in detail. State whether they were prespecified for the review and, if applicable, whether they were primary/main or secondary/additional outcomes. Give the principal measures of effect (such as risk ratio, hazard ratio, difference in means) used for each outcome. | 12-13 |
| Synthesis methods | 14 | Describe the meta-analysis methods used to synthesize IPD. Specify any statistical methods and models  used. Issues should include (but are not restricted to):  • Use of a 1-stage or 2-stage approach  • How effect estimates were generated separately within each study and combined across studies (where applicable)  • Specification of 1-stage models (where applicable) including how clustering of patients within studies  was accounted for  • Use of fixed- or random-effects models and any other model assumptions, such as proportional hazards  • How (summary) survival curves were generated (where applicable)  • Methods for quantifying statistical heterogeneity (such as *I*2 and τ2)  • How studies providing IPD and not providing IPD were analyzed together (where applicable)  • How missing data within the IPD were dealt with (where applicable) | 12-14 |
| Exploration of variation  in effects | A2 | If applicable, describe any methods used to explore variation in effects by study- or participant-level characteristics (such as estimation of interactions between effect and covariates). State all participant-level characteristics that were analyzed as potential effect modifiers and whether these were prespecified. | 12-13 |
| Risk of bias across  studies | 15 | Specify any assessment of risk of bias relating to the accumulated body of evidence, including any pertaining to not obtaining IPD for particular studies, outcomes, or other variables. | Not applicable |
| Additional analyses | 16 | Describe methods of any additional analyses, including sensitivity analyses. State which of these were prespecified. | 14 |
