# Supplementary material for: Efficacy of acupuncture in subpopulations with functional constipation: A protocol for a systematic review and individual patient data meta-analysis
Source: PLoS One. 2022 Apr 12;17(4):e0266075. doi: 10.1371/journal.pone.0266075 (PMC9004736; doi:10.1371/journal.pone.0266075)
Supplement: S1 File — (DOCX) [file pone.0266075.s003.docx]

**Search Strategies:**

| **Database** | **Search strategy** |
| --- | --- |
| Cochrane Central Register of Controlled Trials (CENTRAL), in the Cochrane Library | #1 MeSH descriptor: [Acupuncture] explode all trees  #2 MeSH descriptor: [Acupuncture Therapy] explode all trees  #3 acupunct* or acupress* or acupoint* or electroacupunct* or auriculotherap* or auriculoacupunct* or moxibust* or meridian* or patch*  #4 #1 or #2 or #3  #5 MeSH descriptor: [constipation] explode all trees  #6 constipation or dyschezia or colonic inertia or obstipation or rectal constipation or functional constipation  #7 #5 or #6  #8 #4 and #7 |
| MEDLINE Ovid | 1. exp ACUPUNCTURE/  2. exp ACUPUNCTURE THERAPY/  3. (acupunct* or acupress* or acupoint* or electroacupunct* or auriculotherap* or auriculoacupunct* or moxibust* or meridian* or patch*).mp. [mp=title, abstract, original title, name of substance word, subject heading word, keyword heading word, protocol supplementary concept word, rare disease supplementary concept word, unique identifier, synonyms]  4. 1 or 2 or 3  5. exp constipation/  6. (constipation or dyschezia or colonic inertia or obstipation or rectal constipation or functional constipation).mp. [mp=title, abstract, original title, name of substance word, subject heading word, keyword heading word, protocol supplementary concept word, rare disease supplementary concept word, unique identifier, synonyms]  7. 5 or 6  8. 4 and 7 |
| Scopus | 1. (acupunct* or acupress* or acupoint* or electroacupunct* or auriculotherap* or auriculoacupunct* or moxibust* or meridian* or patch*) in article title, abstract,keywords  2. (constipation or dyschezia or colonic inertia or obstipation or rectal constipation or functional constipation) in article title, abstract,keywords  3. 1 and 2 |
| Embase Ovid | 1. exp acupuncture/  2. (acupunct* or acupress* or acupoint* or electroacupunct* or auriculotherap* or auriculoacupunct* or moxibust* or meridian* or patch*).mp. [mp=title, abstract, heading word, drug trade name, original title, device manufacturer, drug manufacturer, device trade name, keyword, floating subheading word, candidate term word]  3. 1 or 2  4. exp constipation /  5. (constipation or dyschezia or colonic inertia or obstipation or rectal constipation or functional constipation).mp. [mp=title, abstract, heading word, drug trade name, original title, device manufacturer, drug manufacturer, device trade name, keyword, floating subheading word, candidate term word]  6. 4 or 5  7. 3 and 6 |
| Science Citation Index Expanded (Web of Science) | #3 #2 AND #1  #2 TS=(constipation or dyschezia or colonic inertia or obstipation or rectal constipation or functional constipation)  #1 TS=(acupunct* or acupress* or acupoint* or electroacupunct* or auriculotherap* or auriculoacupunct* or moxibust* or meridian* or patch*) |
| Conference Proceedings Citation Index – Science (Web of Science) | #3 #2 AND #1  #2 TS=(constipation or dyschezia or colonic inertia or obstipation or rectal constipation or functional constipation)  #1 TS=(acupunct* or acupress* or acupoint* or electroacupunct* or auriculotherap* or auriculoacupunct* or moxibust* or meridian* or patch*) |
| China Network Knowledge Infrastructure (CNKI) | #1 'constipation' or 'dyschezia' or 'colonic inertia' or 'obstipation' or 'rectal constipation' or ' functional constipation' in abstract  #2 'acupuncture and moxibustion' or 'acupunture' or 'stimulation' or 'electroacupuncture' or 'laser acupuncture' or 'acupressure' or 'acupoint injection' or 'moxibustion' or 'patching' in abstract  #3 random in abstract  #4 randomly grouped in abstract  #5 #3 OR #4  #6 #1 AND #2 AND #5 |
| SinoMed | #1 constipation in abstract  #2 'acupuncture and moxibustion' or 'acupunture' or 'stimulation' or 'electroacupuncture' or 'laser acupuncture' or 'acupressure' or 'acupoint injection' or 'moxibustion' or 'patching' in abstract  #3 random in abstract  #4 randomly grouped in abstract  #5 #3 OR #4  #6 #1 AND #2 AND #5 |
| Wanfang Data | #1 constipation in abstract  #2 'acupuncture and moxibustion' or 'acupunture' or 'stimulation' or 'electroacupuncture' or 'laser acupuncture' or 'acupressure' or 'acupoint injection' or 'moxibustion' or 'patching' in abstract  #3 random in abstract  #4 randomly grouped in abstract  #5 #3 OR #4  #6 #1 AND #2 AND #5 |
| Chongqing VIP (CQVIP) | #1 constipation in abstract  #2 'acupuncture and moxibustion' or 'acupunture' or 'stimulation' or 'electroacupuncture' or 'laser acupuncture' or 'acupressure' or 'acupoint injection' or 'moxibustion' or 'patching' in abstract  #3 random in abstract  #4 randomly grouped in abstract  #5 #3 OR #4  #6 #1 AND #2 AND #5 |
