# Supplementary material for: Efficacy of acupuncture in subpopulations with functional constipation: A protocol for a systematic review and individual patient data meta-analysis
Source: PLoS One. 2022 Apr 12;17(4):e0266075. doi: 10.1371/journal.pone.0266075 (PMC9004736; doi:10.1371/journal.pone.0266075)
Supplement: S1 Appendix — (DOCX) [file pone.0266075.s004.docx]

Search Strategies:

MEDLINE:

(("constipation"[MeSH Terms] OR ((((((("constipation"[Title/Abstract] OR "dyschezia"[Title/Abstract]) OR "colonic inertia"[Title/Abstract]) OR "obstipation"[Title/Abstract]) OR "rectal constipation"[Title/Abstract]) OR "slow transit constipation"[Title/Abstract]) OR "functional constipation"[Title/Abstract]) OR "chronic constipation"[Title/Abstract])) AND ((((("acupuncture"[MeSH Terms] OR "acupuncture therapy"[MeSH Terms]) OR "acupuncture therapy"[MeSH Terms]) OR "acupuncture, ear"[MeSH Terms]) OR "acupuncture points"[MeSH Terms]) OR (((((("acupuncture"[Title/Abstract] OR "acupoint"[Title/Abstract]) OR "tui na"[Title/Abstract]) OR "acupressure"[Title/Abstract]) OR "catgut embedding"[Title/Abstract]) OR "electroacupuncture"[Title/Abstract]) OR "cupping"[Title/Abstract]))) AND ((((((("randomized controlled trial"[Publication Type] OR "controlled clinical trial"[Publication Type]) OR "randomized"[Title/Abstract]) OR "placebo"[Title/Abstract]) OR "clinical trials as topic"[MeSH Terms]) OR "randomly"[Title/Abstract]) OR "trial"[Title]) NOT ("animals"[MeSH Terms] NOT "humans"[MeSH Terms])

Embase:

('constipation'/exp OR (constipation:ti,ab OR dyschezia:ti,ab OR obstipation:ti,ab OR 'rectal constipation':ti,ab OR 'slow transit constipation':ti,ab OR 'functional constipation':ti,ab OR 'chronic constipation':ti,ab)) AND ('acupuncture'/exp OR ('acupuncture':ti,ab OR auriculotherapy:ti,ab OR acupoint:ti,ab OR 'tui na':ti,ab OR acupressure:ti,ab OR 'catgut embedding':ti,ab OR electroacupuncture:ti,ab)) AND ('crossover procedure':de OR 'double-blind procedure':de OR 'randomized controlled trial':de OR 'single-blind procedure':de OR random*:de,ab,ti OR factorial*:de,ab,ti OR crossover*:de,ab,ti OR ((cross NEXT/1 over*):de,ab,ti) OR placebo*:de,ab,ti OR ((doubl* NEAR/1 blind*):de,ab,ti) OR ((singl* NEAR/1 blind*):de,ab,ti) OR assign*:de,ab,ti OR allocat*:de,ab,ti OR volunteer*:de,ab,ti)
